# Density-Independent Mortality and Increasing Plant Diversity Are Associated with Differentiation of *Taraxacum officinale* into r- and K-Strategists

**DOI:** 10.1371/journal.pone.0028121

**Published:** 2012-01-09

**Authors:** Annett Lipowsky, Christiane Roscher, Jens Schumacher, Bernhard Schmid

**Affiliations:** 1 Max Planck Institute for Biogeochemistry, Jena, Germany; 2 Institute of Evolutionary Biology and Environmental Studies, University of Zurich, Zurich, Switzerland; 3 Department of Community Ecology, Helmholtz Centre for Environmental Research, Halle, Germany; 4 Institute of Stochastics, Friedrich Schiller University Jena, Jena, Germany; University of Utah, United States of America

## Abstract

**Background:**

Differential selection between clones of apomictic species may result in ecological differentiation without mutation and recombination, thus offering a simple system to study adaptation and life-history evolution in plants.

**Methodology/Principal Findings:**

We caused density-independent mortality by weeding to colonizer populations of the largely apomictic *Taraxacum officinale* (Asteraceae) over a 5-year period in a grassland biodiversity experiment (Jena Experiment). We compared the offspring of colonizer populations with resident populations deliberately sown into similar communities. Plants raised from cuttings and seeds of colonizer and resident populations were grown under uniform conditions. Offspring from colonizer populations had higher reproductive output, which was in general agreement with predictions of r-selection theory. Offspring from resident populations had higher root and leaf biomass, fewer flower heads and higher individual seed mass as predicted under K-selection. Plants grown from cuttings and seeds differed to some degree in the strength, but not in the direction, of their response to the r- vs. K-selection regime. More diverse communities appeared to exert stronger K-selection on resident populations in plants grown from cuttings, while we did not find significant effects of increasing species richness on plants grown from seeds.

**Conclusions/Significance:**

Differentiation into r- and K-strategists suggests that clones with characteristics of r-strategists were selected in regularly weeded plots through rapid colonization, while increasing plant diversity favoured the selection of clones with characteristics of K-strategists in resident populations. Our results show that different selection pressures may result in a rapid genetic differentiation within a largely apomictic species. Even under the assumption that colonizer and resident populations, respectively, happened to be r- vs. K-selected already at the start of the experiment, our results still indicate that the association of these strategies with the corresponding selection regimes was maintained during the 5-year experimental period.

## Introduction

The evolutionary potential of populations or species is largely determined by the amount and patterns of genetic variation. Natural selection may shape the adaptation of populations in response to local environmental conditions such as disturbance or competition, but little is known about the time-scale of genetic divergence. *Taraxacum officinale* (common dandelion) is a species aggregate with variable ploidy level, mating system and degree of reproductive isolation. However, populations even in strictly apomictic regions can be genetically highly variable [1]. Several studies have shown that different clones in such populations are ecologically differentiated [2], [3]. In a citation classic, Gadgil and Solbrig [2] studied the offspring of four clones or biotypes of *T. officinale* originating from natural successional habitats with different levels of disturbance through trampling and mowing in competition experiments and showed that plants from intensively disturbed habitats had traits expected under r-selection.

According to the biogeographic theory of MacArthur and Wilson [4], [5], populations living in disturbed habitats spend most of their time in exponential growth and should therefore develop traits that allow them to rapidly colonize a habitat and have high intrinsic rates of growth. In other words, they should become r-strategists which are supposed to be particularly successful in unstable environments with density-independent mortality [2], [6] and fluctuating resource availability [7]. In contrast, populations spending most of their time in stationary phase should develop traits that allow them to persist and compete against con-specifics and other species, having high asymptotic population size, hence being K-strategists [5]. A high competitiveness for limiting resources is a prerequisite to stay permanently established in such relatively stable environments with density-dependent mortality and relatively constant resource availability [7]. Because trade-offs may exist between the two strategies, it is expected that no species could be both r- and K-strategist at the same time [2], [6]. Thus, r-strategists may maximize their reproduction and dispersal efforts only at the cost of reduced competitiveness, whereas K-strategist may invest more resources into vegetative biomass and persistence at the cost of reduced reproductive effort [8].

The strongest K-selection may be expected in species-rich habitats with constantly high competition. Several studies in experimental grasslands have shown that increasing species richness is associated with a more complete use of plant-available resources such as light [9], [10] and nutrients [11], thus decreasing the number of available niches. However, effects of species richness as a selection pressure on plant life-history traits and genetic differentiation within populations are poorly understood [12]–[16].

The mechanisms underlying r- and K-strategy selection could be based on genotype selection (i.e., DNA sequence based selection) or, according to recent studies, also on transgenerational non-genetic effects, or on a combination of both. Environmental conditions experienced by parental individuals can affect offspring characteristics such as germination, growth, competitive ability or fecundity [17]. Allocation of maternal resources may improve seed quality and germination, while contrasting results have been found for their relevance during maturity of offspring [18]–[20]. Clonal maternal effects, i.e. if plants are propagated from vegetative plants, are likely to be stronger than maternal effects in plants grown from seeds due to the absence of meiotic barriers, but only few studies have examined patterns of variation between progeny from seeds and vegetative plant parts [21]. Additionally, epigenetic processes, i.e. changes of gene expression without changes in the DNA sequence, such as heritable DNA methylation, may affect offspring phenotype [22].

To test experimentally if r-/K-selection can occur in a species, a first prerequisite is that the species should be genetically polymorphic with regard to the relevant traits under selection. Genetic differentiation within and between plant populations in response to the biotic and abiotic environment is common [23], [24]. Selection pressure on competitive traits should favour the establishment of well-adapted genotypes in relevant timescales [25]. Genotype-specific responses to disturbance and competition have been reported repeatedly from studies in natural systems as well as in experimental systems combining different species [26]–[29]. The particular traits that enable plants to live in disturbed habitats with high density-independent mortality (r-strategy) are similar to those of invading species, e.g. short generation time, large seed number, small seed size and rapid growth [30]–[32]. Traits that enable plants to live in less variable environments with density-dependent mortality (K-strategy) are those of good survivors and competitors, e.g. high investment in root biomass and individual seed mass [33], [34].

Here we revisited the question about r- and K-selection in *T. officinale* using a grassland biodiversity experiment in Jena, Germany [35]. Gadgil and Solbrig [2] compared in their study plants obtained from different habitats, which presumably differed in density-independent mortality regimes. In contrast to their approach, we deliberately created r- and K-selection regimes ourselves. Our controlled r-selection regime was imposed for a period of 5 years by weeding *T. officinale* (causing density-independent mortality) in plots of varying species richness, where it did not belong to the sown species combinations and was considered as an invader. We compared this treatment with a K-selection regime, in which *T. officinale* was sown as a resident species in the plots of increasing plant species richness and was not weeded during the 5 years. After the 5 years of selection, we grew offspring from seeds and cuttings of both types of plants under uniform conditions in a common garden.

We hypothesized that 5 years of differential experimental selection resulted in a genetic differentiation among dandelion populations. If this would happen, it could be due to differential selection among co-occurring clones because *T. officinale* is largely apomictic. An alternative possibility would be differential maternal effects which could be larger in cutting- than in seed-derived material. The offspring of r-selected *T. officinale* should show the traits expected for r-strategists, in particular increased reproductive output, higher shoot:root ratio and fast reproduction, and the offspring of K-selected *T. officinale* should show traits expected for K-strategists, in particular increased allocation to vegetative growth and increased plant and propagule size. In addition, because we assumed that increasing inter-specific competition in plant communities of higher species richness exerts stronger K-selection than do species-poor communities, we hypothesized that these traits expected for K-strategists should be positively selected with increasing species richness of plots from which K-selected offspring were collected.

## Results

### Differences between r- and K-selected plants

**Figure 1:**
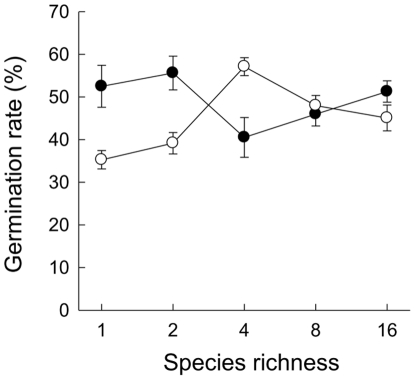
Species richness effects on germination rates of seeds of *T. officinale*. Material was collected from colonizer populations (open circles) and resident populations (closed circles) in the Jena Experiment, 5 years after sowing. Values are means per species-richness level ±1 SE.

Standard germination tests with seeds revealed considerable variation in germination rates among maternal plants. Seeds of 14% of maternal plants either failed to germinate completely or had germination rates below 10%; seeds of 28% of maternal plants had germination rates above 70%. Average germination rates of seeds from r-selected (i.e. weedy, colonizing) and K-selected (i.e. resident) maternal plants did not differ significantly (Fig. 1; Table 1).

**Table 1:** Mixed-effects model analysis of characteristics measured on plants grown from cuttings and seeds of resident and colonizer populations of *T. officinale* in the Jena Experiment, 5 years after sowing.

	Germination (%)		Shoot biomass		Root biomass		Shoot:root ratio		Vegetative biomass		Reproductive biomass	
	L ratio	p	L ratio	p	L ratio	p	L ratio	p	L ratio	p	L ratio	p
Seedlings (S) vs. cuttings (C)	n.a.	n.a.	**13.88**	**<0.001**	n.a.	n.a.	n.a.	n.a.	**18.23**	**<0.001**	0.02	0.891
Disturbance (rK)	1.93	0.165	0.23	0.628	**16.12**	**<0.001**	**14.72**	**<0.001**	0.85	0.358	0.48	0.490
Species richness (log SR)	**5.30**	**0.021**	**9.19**	**0.002**	<0.01	0.964	0.02	0.9019	**4.89**	**0.027**	**5.51**	**0.019**
rK x log SR	1.57	0.210	3.67	0.055	1.09	0.297	<0.01	1.000	2.69	0.101	**4.34**	**0.037**
S vs. C x rK	n.a.	n.a.	0.24	0.626	n.a.	n.a.	n.a.	n.a.	1.17	0.280	1.14	0.287
S vs. C x log SR	n.a.	n.a.	0.01	0.912	n.a.	n.a.	n.a.	n.a.	0.04	0.844	0.41	0.520

	Number of leaves		Maximum leaf length		Number of flower heads		Flower head diameter		Seed mass per flower head			Seed mass per plant	
	L ratio	p	L ratio	p	L ratio	p	L ratio	p	L ratio	p	L ratio		p
Seedlings (S) vs. cuttings (C)	0.39	0.530	**6.38**	**0.012**	2.80	0.095	n.a.	n.a.	**5.74**	**0.017**	**4.02**		**0.045**
Disturbance (rK)	**5.40**	**0.020**	0.02	0.876	**23.93**	**<0.001**	2.70	0.100	**16.50**	**<0.001**	2.26		0.133
Species richness (log SR)	1.11	0.293	2.10	0.148	0.89	0.346	3.30	0.069	1.19	0.276	2.52		0.112
rK x log SR	1.07	0.301	1.41	0.235	**4.48**	**0.034**	**4.73**	**0.030**	1.23	0.268	**5.86**		**0.016**
S vs. C x rK	3.19	0.074	0.06	0.807	2.86	0.091	n.a.	n.a.	0.10	0.755	0.78		0.378
S vs. C x log SR	2.04	0.153	2.56	0.110	<0.01	0.947	n.a.	n.a.	0.03	0.865	0.10		0.758

	Mean seed mass		Seed number per plant		First day of flowering		Day of seed maturity		Height of the flower stalk	
	L ratio	p	L ratio	p	L ratio	p	L ratio	p	L ratio	p
Seedlings (S) vs. cuttings (C)	**19.69**	**<0.001**	<0.01	0.981	n.a.	n.a.	2.29	0.130	0.41	0.523
Disturbance (rK)	**18.94**	**<0.001**	**13.69**	**<0.001**	**11.29**	**<0.001**	2.57	0.109	0.87	0.351
Species richness (log SR)	0.03	0.855	**4.00**	**0.046**	0.47	0.493	0.70	0.402	0.19	0.659
rK x log SR	**5.71**	**0.017**	1.76	0.184	1.13	0.287	0.35	0.552	0.05	0.820
S vs. C x rK	**17.53**	**<0.001**	**6.72**	**0.010**	n.a.	n.a.	**11.31**	**<0.001**	**20.30**	**<0.001**
S vs. C x log SR	0.03	0.871	0.12	0.725	n.a.	n.a.	0.51	0.477	0.17	0.682

Note: Models were fitted by stepwise inclusion of model terms. Listed are the results of likelihood-ratio tests that were applied to assess model improvement (L ratio) and the statistical significance of these tests (p values).

**Figure 2:**
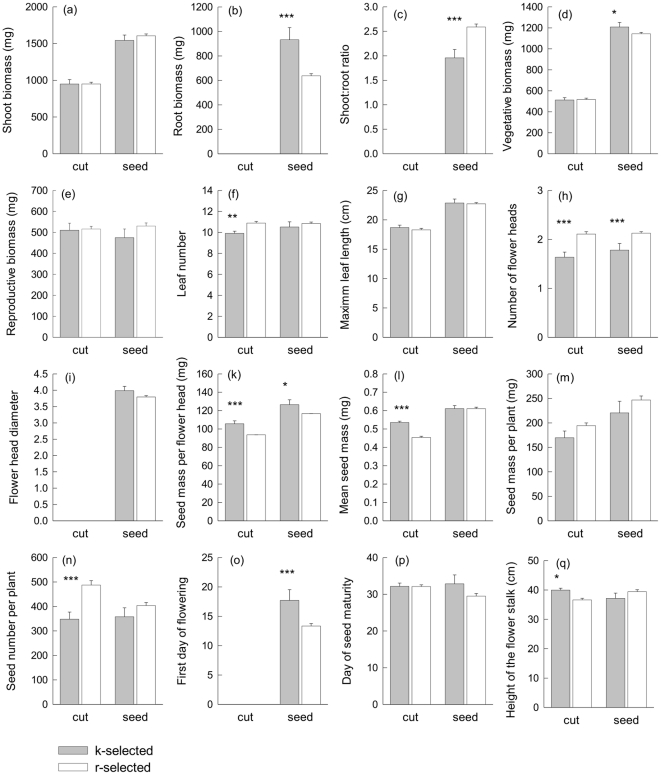
Effects of r- and K-selection regime on plant characteristics of *T. officinale*. Shoot biomass (a), root biomass (b), shoot:root ratio (c), aboveground vegetative biomass ( = leaves) (d), reproductive biomass (e), leaf number (f), maximum leaf length (g), flower head number (h), flower head diameter (i), seed mass per flower head (k), average seed mass (l), seed mass per plant individual (m), seed number per plant individual (n), first day of flowering (o), first day of seed maturity (p), and flower stalk length at seed maturity (q) were measured in plants grown from cuttings and seeds of resident populations (grey bars) and colonizer populations (white bars) of *T. officinale* in the Jena Experiment, 5 years after sowing. Values are means across populations ±1 SE for plants grown from cuttings and from seeds.

Shoot biomass also did not differ significantly between r- and K-selected populations, independently of whether plants were grown from seeds or from cuttings (Fig. 2a; Tables 1, S1). However, root biomass of plants derived from seeds was higher in K-selected than r-selected populations, which consequently also had higher shoot:root ratios (Fig. 2b, c; Tables 1). K-selected plants derived from seeds had a higher vegetative biomass at the cost of reduced reproductive biomass compared with r-selected plants derived from seeds (Fig. 2d, e; Table S1). On average, r-selected plants produced more leaves (of similar length) than K-selected plants (Fig. 2f, g; Tables 1) and this difference was significant for plants derived from cuttings (Table S1).

In total, 84% of all cuttings and 75% of all plants grown from seeds produced flowers at harvest time. K-selected plants were less likely to flower (74% of K-selected vs. 82% of r-selected plants) and produced fewer flower heads than did r-selected plants (Tables 1), independently of whether they were grown from seeds or from cuttings (Fig. 2h; Table S1). r-selected plants also had a lower seed mass per flower head than did K-selected plants (Fig. 2k; Tables 1, S1). Nevertheless, due to smaller average seed mass (Fig. 2l, Table 1) and the higher number of flower heads, r-selected plants produced more seeds per plant (Fig. 2n, Table 1). Significant interaction terms between propagation method (cuttings vs. seed-derived plants) and selection regime (r vs. K) suggested that effects of the selection regime on reproductive traits differed to some degree between cuttings and seedlings. Mean seed mass was reduced and seed number per plant was strongly increased in cuttings of r-selected plants (Table S1), whereas these differences among selection regimes were not significant for plants grown from seeds (Table S1).

Finally, r-selected plants also showed more precocity (Table 1): r-selected plants grown from seeds flowered on average 4.4 days earlier (Fig. 2o) and produced mature seeds 3.3 days earlier than K-selected plants grown from seeds (Fig. 2p; Table S1). K-selected plants grown from cuttings had a longer flower stalk than r-selected plants grown from cuttings (Fig. 2q; Table S1).

### Selection response to biodiversity

Average germination rates of seed material collected in the biodiversity experiment increased with increasing species richness (Fig. 1; Table 1). High species richness selected for *T. officinale* plants of large size (large shoot and reproductive biomass, high seed mass per flower head), but significant effects of increasing species richness were restricted to plants grown from cuttings (Figs. S1, S2, S3; Tables 1, S1). Reproductive biomass, seed mass per plant and number of flower heads showed a stronger selection response to biodiversity in K-selected plants (significant interaction term “rK × log SR”, Fig. S1 c, f, i; Table S1), although the measured values were higher in r-selected plants.

### Differences between cuttings and plants grown from seeds

Apart from the somewhat different responses to r-and K-selection, the average plant raised from cuttings also differed from the average plant raised from seeds in a number of vegetative (e.g. shoot biomass, vegetative biomass, leaf length) and reproductive traits (Fig. 2; Table 1). Cuttings were generally lighter, produced shorter leaves and had a lower mean seed mass and a lower seed mass per flower head and per plant than plants grown from seeds.

## Discussion

### r- and K-selection in *Taraxacum officinale*

In the present study we wanted to assess whether 5 years of density-independent mortality due to regular weeding and density-dependent mortality caused by competitive interactions in plant communities of different species richness can cause differentiation between colonizer and resident populations of *T. officinale*. Based on a common garden experiment with offspring raised from cuttings or seeds of these populations, we have shown for a number of traits characterizing biomass allocation and reproductive output that density-independent mortality (r-selection) caused different adaptive responses than density-dependent mortality (K-selection). In contrast to a study by Molgaard [36], not only morphological plasticity in response to different disturbance regimes (mowing height), but presumably also heritable, genetic or epigenetic differentiation between the differently selected *T. officinale* populations was found in our experiment, as indicated by differential effects of the r- and K-selection regime on the seed-derived progeny. The results of our study show that the concept of r- and K-strategy [2], [5], [6] is applicable to the strategies developed in populations of *T. officinale* in response to different selection regimes over a 5-year period. These two opposite strategies [2], [30] seem to be adapted in one extreme to unstable environments where resource fluctuations and density-independent mortality are major selection pressures (r-selection) and in the other extreme to stable environments where habitat carrying capacity, competitive interactions and density-dependent mortality are major selection pressures (K-selection). Natural selection should drive a trade-off between both strategies and maximize either the success of r- or K-strategists under these contrasting selection regimes.

In our common garden experiment, plants originating from repeatedly weeded colonizer populations showed the expected characteristics of r-strategists. These plants, compared with resident plants, had larger shoot:root ratios and invested a greater proportion of their available resources into a maximization of reproductive output, i.e. they produced a higher number of flower heads and a larger number of seeds. At the same time seed mass of these plants was lower, presumably enhancing wind-dispersal [37] and thus increasing colonization potential [38]. All these features, including faster reproduction, are reported as common life-history traits not only of r-strategists, but also as typical characteristics of weedy or invasive species [39], [40].

In contrast, plants originating from resident populations needing more time for flowering and seed maturation and producing larger seeds with higher germination rates fit the profile expected for K-selected species [6]. Heavier achenes are more likely to germinate successfully in *T. officinale* [41] and correlate positively with post-germination performance such as seedling mass [42]. The resident populations also allocated a greater proportion of their biomass to roots; and a lower shoot:root ratio is frequently correlated with a higher plant tolerance to competition, drought or frost [43], [44]. This feature is not only reported as a typical life-history trait of K-strategists, but also included in concepts developed for differences between early vs. late-successional species, where the latter are also expected to experience intense competition [45]. However, because the applied selection pressures of density-independent mortality vs. competition are explicitly specified as the underlying mechanisms of r- vs. K-selection, we prefer to put our study into this perspective. *Taraxacum officinale* subjected for 5 years to r-selection showed a shortened time to reproduction and had a higher reproductive output, i.e. the plants were optimized for rapid population growth. The same species subjected for 5 years to K-selection invested more biomass into roots and increased individual seed mass.

### High species richness enhances K-selection

We observed that resident populations after 5 years showed significant differentiation of K-strategy traits in response to the species richness gradient present in the biodiversity field experiment. This indicates that diverse experimental communities imposed stronger K-selection on the residents than did less diverse communities. However, the differentiation in response to increasing species richness was largely restricted to plants grown from cuttings and therefore at least in part presumably attributable to maternal effects. It is likely that the more diverse experimental communities with higher plant densities and community biomass [46] have exerted a stronger competitive pressure on the *T. officinale* plants. That the colonizer populations did not show this response to increasing community diversity is consistent with the fact that they were r-selected by the density-independent weeding and thus did not have a chance to experience the increased density-dependent competition along the diversity gradient for long enough.

### Differences between cuttings and plants grown from seeds

In our study, differences between cuttings and plants grown from seeds were found in vegetative biomass, maximum leaf length and seed output (seed mass per flower head and plant, average seed mass; Table 1). This variation was presumably due to different starting capital and different soil substrate provided to cuttings and seed-derived plants. Cuttings and offspring from seeds did not differ in traits assumed to be less influenced by starting capital and soil nutrients, e.g. the number of leaves and the number of flower heads. However, there was some indication that the selection regime caused by density-independent mortality by weeding had more pronounced effects on cuttings, i.e. reduction in mean seed mass, increase in seed number per plant and number of leaves, was stronger and statistically significant in r-selected plants grown from cuttings compared to those cultivated from seeds (Fig. 2).

However, significant effects of the r- and K-selection regime on several vegetative and reproductive traits measured on plants derived from seeds (see Table S1) suggested that our results were probably caused by genetic selection (i.e. different DNA sequences) together with maternal effects. In our experiment, we cannot differentiate between these two possibilities. We also cannot differentiate between various possible types of maternal effects which may include the maternal provision of resources, hormones, enzymes or toxins to the seed or epigenetic mechanisms such as DNA methylation causing stable alterations in gene activity [17], [22]. Only recently, it has been shown that heritable DNA methylation is common in apomictic dandelion and might contribute significantly to the evolutionary potential of apomictic lineages with limited genetic variation [47].

### Caveats

In our study we assumed that colonizer as well as resident populations represent a mixture including genotypes from the originally sown seed material (probably the major component of the mixture) and invading genotypes from the surroundings of the experimental site (probably the minor component of the mixture). One, in our view unlikely assumption to explain our results would be that resident populations were more or less exclusively derived from sown seeds *and* that these seeds originated from already K-selected populations whereas the colonizer populations were more or less exclusively derived from seeds dispersing from local populations *and* that these were already r-selected. The reason why we think this explanation is unlikely is that the seeds for the establishment of the biodiversity experiment were collected in natural habitats of different successional age and with diverse regimes of disturbance and competition (fallow land, established grasslands). Similarly, seed sources in the near surroundings of the Jena experimental site comprise established grasslands, agricultural land and ruderal sites. A high clonal diversity with few clones widespread and many clones restricted to single populations is typical for the genetic population structure of *T. officinale* [1], [48]. Nevertheless, even if a “pre-adaptation of K-strategists” in resident populations and “pre-adaptation of r-strategists” in colonizer populations would explain our results, it would still show that 5 years of deliberate r- and K-selection kept the original populations apart. Additionally, the effects of species richness on the degree of K-selection among resident populations is even less likely to be due to such biased mixing of differently but “correctly pre-adapted” seed populations.

### Conclusions

In this study we have investigated whether a short period of 5 years experimentally controlled selection may result in a heritable genetic differentiation among populations of the largely apomictic species *T. officinale*. More than 35 years ago Gadgil and Solbrig [2] reported in a classic comparative study that plants of *T. officinale* originating from sites with presumably different selection regimes over an unknown period had developed traits expected under r- or K-selection. In our experimental approach we created the r- and K-selection regimes ourselves by weeding (density-independent mortality, r-selection) and increasing inter-specific competition along a species-richness gradient (K-selection). After 5 years, these selection regimes had resulted in plants that showed the corresponding traits expected under r- and K-selection in a common environment. The differential response to increasing species richness was only found in plants derived from cuttings suggesting strong maternal effects. However, it is probable that the response to the r- and K-selection regime was not exclusively due to maternal effects. It is most likely that differential mortality and asexual reproduction of different clones explain this rapid differentiation, while recombination or mutation events were probably of minor importance [1]. In this respect *T. officinale* represents a simple system to study adaptive evolution of life-history traits in plants. A similar selection experiment has recently been carried out between inbred lines of the model plant *Arabidopsis thaliana* [49], but we did not find further studies done with wild plants.

## Materials and Methods

### Study species

*Taraxacum officinale* Wiggers (Asteraceae) is a herbaceous perennial plant species commonly found in pastures, lawns, along roadsides and in ruderal places. The vegetative plant is characterized by a leaf rosette with a taproot. The species flowers mainly in spring (late April to May). The genus *Taraxacum* contains a complex of diploid sexual and polyploid apomictic forms. Although the plants produce conspicuous flower heads with a copious amount of nectar and pollen, seeds are often produced asexually by diplospory [50]. The seeds disperse through wind or adhesion, often near to the parent plant [51]. In the study region, *T. officinale* is an apomictic, triploid species with almost exclusively asexual reproduction. Numerous agamospecies of *T. officinale* commonly coexist within communities [52]–[54].

The species has a near cosmopolitan distribution. *Taraxacum officinale* is an invasive (naturalized) weed across North America [55] and Japan [56]; no sexual reproduction was discovered there [9], [56], [57]. The morphological variability often observed in populations of *T. officinale* has both a genetic [9], [28], [58] and a plastic [36] component. Despite their predominant asexual reproduction, some single maternal plants have been observed to generate heritable variation among their offspring [51], [59].

### Selection experiment

This study was implemented as a part of the Jena Experiment, a large integrated biodiversity experiment in Germany [35]. The experimental site is located in the floodplain of the river Saale near the city of Jena (50°55′ N, 11°35′ E, 130 m a.s.l.). The area around Jena has a mean annual air temperature of 9.3°C and mean annual precipitation is 587 mm [60]. In total, the Jena Experiment has 78 plots of 20×20 m size that cover a gradient in species richness (1, 2, 4, 8 to 16) and functional group richness (1–4; legumes, grasses, tall herbs, small herbs). The different mixtures were determined by random drawings with replacement from a pool of 60 species typical of species-rich, semi-natural grasslands (for details see [35]). All species were also grown in monoculture on plots of 3.5×3.5 m size. Because of a gradient in soil characteristics across the experimental site all plots were grouped into four blocks parallel to the river. The full experiment was established by sowing in spring 2002. All experimental plots were mown twice a year (early June, September) as usual for extensive hay meadows in the region and did not receive any fertilizer. Unwanted species, i.e. species not included in the originally sown plant communities, were removed twice every year in April and July.

Seeds were purchased from a commercial supplier (Rieger-Hofmann GmbH, Blaufelden-Raboldshausen, Germany). Seeds of *T. officinale* used to establish the biodiversity experiment were a mixture collected from natural populations in established grasslands and fallow land in Southern Germany (pers. comm. E. Rieger) and were therefore likely to represent a large number of genotypes adapted to different environments. *Taraxacum officinale* was included in the experimental species pool and belonged to the community of originally sown ( = resident) species in 12 out of 78 large plots and 1 small monoculture. In addition, this species disseminated spontaneously from within and around the experimental plots and colonized all other plots where it had not been sown from the beginning of the biodiversity experiment ([61], Table S2). In these plots, all plants of *T. officinale* were removed during the biannual weeding campaigns to maintain the originally sown species combinations (see above), while in resident populations there was no weeding at all of *T. officinale*. Therefore, the full duration of the 5-year differential selection regime applied to both resident and colonizing populations. Although the weedy plants of *T. officinale* were pulled out with their roots or cut below the leaf rosette in case they could not be pulled out with roots, they could continually re-establish after the density-independent mortality caused by the weeding, probably by both re-grow from root stumps and re-colonization from seeds. In addition, *T. officinale* usually flowers during the first weeding period (in April); thus weedy individuals also occasionally set seeds and distribute them before weeding. Seeds of *T. officinale* mostly germinate in summer after first mowing, and therefore surviving seedlings may establish and eventually reproduce in spring until the early-season weeding in the next growing season [62].

### Experiment to test selection response

#### Seedlings

Seeds of randomly chosen maternal plants of *T. officinale* were collected in all 78 large plots of the biodiversity experiment and in a small plot with a *T. officinale* monoculture in May 2007. The seeds of 20 maternal plants were collected in every plot where *T. officinale* belonged to the originally sown species ( = 13 resident populations, Table S2). Although some of these plants may have colonized later via seeds from outside, we treat them all as resident plants that we assume to have experienced a K-selection regime (high competition, low density-independent mortality; [2], [6]). The seeds of 10 maternal plants were collected in every remaining large plot where *T. officinale* occurred as a weed ( = 66 colonizer populations, Table S2). These plants were all considered as weedy plants that experienced an r-selection regime (high density-independent mortality).

Seeds were stored at 4°C until 30 seeds per individual were plated for germination in Petri dishes on moistened filter paper in an unheated glasshouse in early September 2007. Seedlings were counted fortnightly after sowing to determine germination rates. Three seedlings per maternal plant were transplanted into QuickPot™ trays (cylindrical pots with a volume of 0.2 L, Herku-Plast, Germany) filled with compost (GEMES, Germany). Plants were grown in an unheated glasshouse without artificial light. In mid-October plantlets from the QuickPot™ trays were transplanted into 1-L flower pots filled with compost (GEMES, Germany). Pots were buried in a garden bed and covered with garden fleece to avoid frost damage in winter. Plants of one experimental block (block 4) were destroyed by mice during this period. From mid-February 2008 onwards all remaining plants were cultivated in two glasshouse chambers. Plants were arranged in eight blocks (four for plants grown from cuttings and four for plants grown from seeds) different to the experimental blocks of the field experiment. Temperatures ranged from 10–15°C during the day and ≥5°C at night. Additional light was provided by high-pressure potassium lamps (SON-T AGRO 400 W, Philipps GmbH, Germany) 3 h per day to initiate flower formation. Temperature was increased to 15–22°C at day and 5–10°C at night on 17 March 2008 to simulate spring.

#### Cuttings

In March 2007, *T. officinale* cuttings were taken in all large plots of the biodiversity experiment and a small monoculture. All cuttings had between 2–5 leaves and a height of 1–5 cm. Cuttings with a 5 cm long root were made with a blade. Again, we collected 20 plants from the 13 plots where *T. officinale* was a resident species including the small monoculture, and 10 plants from the 66 plots where *T. officinale* was a weed.

Cuttings were planted into QuickPot™ trays filled with a mixture of quartz sand and soil from the experimental site (1∶1) and placed outside the glasshouse on a vegetation-free area. In April 2007, the cuttings were planted into 1-L flower pots filled with the same soil mixture. The plants were fertilized biweekly with 200 ml of a 0.2% compound fertilizer (8∶8∶6 N∶P∶K, Wuxal Super, Monheim, Germany) to avoid nutrient limitation. Similar to plants raised from seeds all plants raised from cuttings, were returned to the glasshouse in mid-February 2008, blocked according to the experimental blocks of the field experiment and grown under the same conditions as described for plants grown from seeds.

#### Measurements

Plant individuals were monitored daily for the onset of flowering. The following measurement and harvesting scheme was used (Table S3). The first flowering offspring of each maternal plant grown from seeds was harvested when the first flower head was completely open. The second and third offspring of each maternal plant grown from seeds and the cuttings were harvested when the first capitulum produced ripe seeds. Harvest covered a period of 5 weeks starting on 1 March 2008. All plants that did not produce any buds or flower heads were harvested on 4–5 April 2008. Leaf number, number of flower heads and the length of the longest leaf were recorded on all individuals. Flower head diameter and root biomass were only measured for the first offspring of each maternal plant grown from seeds. For the second offspring of each maternal plant grown from seeds and for the cuttings, seeds of the first capitulum that produced ripe seeds were collected and seed mass was determined. Seed mass per plant was estimated as seed mass per flower head multiplied by the number of flower heads. Additionally, a bulk sample with 30 seeds was weighed to determine average seed mass. Seed number per plant was assessed by dividing seed mass per plant by average seed mass. The dry mass of all plant parts, i.e. shoots, roots, inflorescences and seeds was determined after drying at 70°C (48 h). In total, 2577 plants were harvested: 989 plants raised from cuttings and 1588 plants raised from seeds.

### Statistical analyses

Mixed-effects models with maximum likelihood (ML) estimation of fixed effects and variance components (software R, version 2.9.0, R Development Core Team 2007, http://www.R-project.org; package *lme4*; [63]) were used for data analysis because of the unbalanced design (different numbers of plots and plants for the resident and colonizer populations) and the multiple random terms in the model [64]. Blocks of the greenhouse and blocks and plots of the biodiversity experiment were considered as random terms. Family ( = maternal plant in case of seedlings) was included as an additional random term when traits were measured on several offspring per maternal plant. The fixed terms were main selection regime (K- vs. r-selection), biodiversity selection regime (species richness of the experimental communities as log-linear term (log SR) and presence/absence of each of the four functional groups in the experimental communities) and interactions of the previous terms. These fixed terms were added stepwise to the models. Likelihood-ratio tests were applied to compare the models obtained in the different steps and to assess the significance of the fixed effects (Appendix S1). Because the presence/absence of particular functional groups in the experimental communities had only marginal effects in this study, we removed them from the final models. Firstly, data were analysed combining plants grown from seeds and grown from cuttings. Because we found significant differences in a number of measured traits between plants grown from cuttings and seeds in response to the experimental factors (r- vs. K-selection regime, species richness of the experimental communities), we secondly performed separate analyses for data based on cuttings and seedlings. If necessary, data were log-transformed and arcsine square-root transformed in case of germination rates prior to the analyses to meet the assumptions of normality and variance homogeneity.

## Supporting Information

Figure S1 **Species richness effects on biomass, vegetative and reproductive plant characteristics of** ***T. officinale*** **grown from cuttings.** Shoot biomass (a), aboveground vegetative biomass ( = leaves) (b), reproductive biomass (c), leaf number (d), maximum leaf length (e), flower head number (f), seed mass per flower head (g), average seed mass (h), seed mass per plant individual (i), seed number per plant individual (k), first day of seed maturity (l), and flower stalk length at seed maturity (m) were measured in plants grown from cuttings of colonizer populations (open circles) and resident populations (closed circles) of *T. officinale* in the Jena Experiment, 5 years after sowing. Values are means per species-richness level ±1 SE.
(DOC)

Figure S2 **Species richness effects on biomass and vegetative plant characteristics of** ***T. officinale*** **grown from seeds.** Shoot biomass (a), root biomass (b), shoot:root ratio (c), aboveground vegetative biomass ( = leaves) (d), reproductive biomass (e), leaf number (f), and maximum leaf length (g) were measured in plants grown from seeds of colonizer populations (open circles) and resident populations (closed circles) of *T. officinale* in the Jena Experiment, 5 years after sowing. Values are means per species-richness level ±1 SE.
(DOC)

Figure S3 **Species richness effects on reproductive plant characteristics of** ***T. officinale*** **grown from seeds.** Flower head number (a), flower head diameter (b), seed mass per flower head (c), seed mass per plant individual (d), average seed mass (e), seed number per plant individual (f), first day of flowering (g), first day of seed maturity (h), and flower stalk length at seed maturity (i) were measured in plants grown from seeds of colonizer populations (open circles) and resident populations (closed circles) of *T. officinale* in the Jena Experiment, 5 years after sowing. Values are means per species-richness level ±1 SE.
(DOC)

Table S1 Separate mixed-effects model analysis of characteristics measured on plants grown either from cuttings or from seeds of resident and colonizer populations of *T. officinale* in the Jena Experiment, 5 years after sowing.
(DOC)

Table S2 Origins of plants grown from cuttings and seeds of *Taraxacum officinale* from experimental plots sown with a different number of species.
(DOC)

Table S3 Measured traits of plants grown from seeds or cuttings.
(DOC)

Appendix S1 The R code for statistical analysis with mixed-effects models.
(DOC)
