# Supplementary material for: Density-Independent Mortality and Increasing Plant Diversity Are Associated with Differentiation of Taraxacum officinale into r- and K-Strategists
Source: PLoS One. 2012 Jan 9;7(1):e28121. doi: 10.1371/journal.pone.0028121 (PMC3253783; doi:10.1371/journal.pone.0028121)
Supplement: Table S1 — Separate mixed-effects model analysis of characteristics measured on plants grown either from cuttings or from seeds of resident and colonizer populations of T. officinale in the Jena Experiment, 5 years after sowing. (DOC) [file pone.0028121.s004.doc]

**Table S1**

|  | Shoot biomass | |  |  |  | Root biomass | |  |  |  | Shoot:root ratio | |  |  |  | Vegetative biomass | | |  |
| --- | --- | --- | --- | --- | --- | --- | --- | --- | --- | --- | --- | --- | --- | --- | --- | --- | --- | --- | --- |
|  | Cuttings | | Seedlings | |  | Cuttings | | Seedlings | |  | Cuttings | | Seedlings | |  | Cuttings | | Seedlings | |
|  | L ratio | p | L ratio | p |  | L ratio | p | L ratio | p |  | L ratio | p | L ratio | p |  | L ratio | p | L ratio | p |
| Disturbance (rK) | 0.01 | 0.935 | 0.06 | 0.810 |  | n.a. | n.a. | **16.12** | **<0.001** |  | n.a. | n.a. | **14.72** | **<0.001** |  | 0.23 | 0.632 | **4.99** | **0.025** |
| Species richness (log SR) | **4.47** | **0.035** | 1.91 | 0.166 |  | n.a. | n.a. | <0.01 | 0.964 |  | n.a. | n.a. | 0.02 | 0.902 |  | 0.76 | 0.385 | 1.08 | 0.298 |
| rK x log SR | **4.03** | **0.045** | 0.50 | 0.481 |  | n.a. | n.a. | 1.09 | 0.297 |  | n.a. | n.a. | <0.01 | 1.000 |  | 2.00 | 0.157 | 0.43 | 0.514 |
|  |  |  |  |  |  |  |  |  |  |  |  |  |  |  |  |  |  |  |  |
|  | Reproductive biomass | | |  |  | Number of leaves | | |  |  | Maximum leaf length | | |  |  | Number of flower heads | | |  |
|  | Cuttings | | Seedlings | |  | Cuttings | | Seedlings | |  | Cuttings | | Seedlings | |  | Cuttings | | Seedlings | |
|  | L ratio | p | L ratio | p |  | L ratio | p | L ratio | p |  | L ratio | p | L ratio | p |  | L ratio | p | L ratio | p |
| Disturbance (rK) | 0.01 | 0.913 | 2.25 | 0.134 |  | **11.66** | **0.001** | 0.08 | 0.783 |  | <0.01 | 0.983 | 0.16 | 0.687 |  | **17.14** | **<0.001** | **11.49** | **<0.001** |
| Species richness (log SR) | **3.88** | **0.049** | 2.22 | 0.136 |  | 0.01 | 0.943 | 0.98 | 0.322 |  | 0.05 | 0.819 | 3.43 | 0.064 |  | 0.58 | 0.446 | 0.46 | 0.496 |
| rK x log SR | **4.06** | **0.044** | 0.67 | 0.413 |  | 0.10 | 0.754 | 0.33 | 0.566 |  | 0.56 | 0.454 | 1.45 | 0.229 |  | **3.80** | **0.049** | 0.70 | 0.402 |
|  |  |  |  |  |  |  |  |  |  |  |  |  |  |  |  |  |  |  |  |
|  | Flower head diameter | | |  |  | Seed mass per flower head | | | |  | Mean seed mass | | |  |  | Seed mass per plant | | |  |
|  | Cuttings | | Seedlings | |  | Cuttings | | Seedlings | |  | Cuttings | | Seedlings | |  | Cuttings | | Seedlings | |
|  | L ratio | p | L ratio | p |  | L ratio | p | L ratio | p |  | L ratio | p | L ratio | p |  | L ratio | p | L ratio | p |
| Disturbance (rK) | n.a. | n.a. | 2.70 | 0.100 |  | **12.09** | **<0.001** | **5.48** | **0.019** |  | **28.24** | **<0.001** | 0.07 | 0.798 |  | 2.17 | 0.141 | 0.26 | 0.611 |
| Species richness (log SR) | n.a. | n.a. | 3.30 | 0.069 |  | 1.00 | 0.317 | 0.04 | 0.851 |  | 0.20 | 0.657 | 0.07 | 0.793 |  | 1.29 | 0.257 | 0.83 | 0.363 |
| rK x log SR | n.a. | n.a. | **4.73** | **0.030** |  | 1.78 | 0.182 | 0.63 | 0.428 |  | 3.33 | 0.068 | 1.41 | 0.240 |  | **5.80** | **0.016** | 0.27 | 0.600 |
|  |  |  |  |  |  |  |  |  |  |  |  |  |  |  |  |  |  |  |  |
|  | Seed number per plant | | |  |  | First day of flowering | | |  |  | First day of seed maturity | | | |  | Height of the flower stalk | | | |
|  | Cuttings | | Seedlings | |  | Cuttings | | Seedlings | |  | Cuttings | | Seedlings | |  | Cuttings | | Seedlings | |
|  | L ratio | p | L ratio | p |  | L ratio | p | L ratio | p |  | L ratio | p | L ratio | p |  | L ratio | p | L ratio | p |
| Disturbance (rK) | **13.97** | **<0.001** | 0.16 | 0.687 |  | n.a. | n.a. | **11.29** | **<0.001** |  | 0.20 | 0.656 | 3.55 | 0.060 |  | **6.04** | **0.014** | 2.44 | 0.119 |
| Species richness (log SR) | 1.56 | 0.212 | 0.86 | 0.354 |  | n.a. | n.a. | 0.47 | 0.493 |  | <0.01 | 0.972 | 0.47 | 0.494 |  | <0.01 | 0.987 | 0.13 | 0.716 |
| rK x log SR | 2.34 | 0.126 | 0.03 | 0.856 |  | n.a. | n.a. | 1.13 | 0.287 |  | 2.05 | 0.152 | 0.33 | 0.568 |  | 0.38 | 0.537 | 0.18 | 0.668 |

Note: Models were fitted by stepwise inclusion of model terms. Listed are the results of likelihood-ratio tests that were applied to assess model improvement (L ratio) and the statistical significance of these tests (p values).
