# Supplementary material for: Density-Independent Mortality and Increasing Plant Diversity Are Associated with Differentiation of Taraxacum officinale into r- and K-Strategists
Source: PLoS One. 2012 Jan 9;7(1):e28121. doi: 10.1371/journal.pone.0028121 (PMC3253783; doi:10.1371/journal.pone.0028121)
Supplement: Table S2 — Origins of plants grown from cuttings and seeds of Taraxacum officinale from experimental plots sown with a different number of species. (DOC) [file pone.0028121.s005.doc]

**Table S2**

|  |  |  | Plants grown from seeds | |  | Plants grown from cuttings | |
| --- | --- | --- | --- | --- | --- | --- | --- |
|  |  |  | Resident populations | Colonizer populations |  | Resident populations | Colonizer populations |
| Species richness | 1 |  | 1 | 12 |  | 1 | 16 |
|  | 2 |  | 2 | 10 |  | 2 | 14 |
|  | 4 |  | 1 | 11 |  | 1 | 15 |
|  | 8 |  | 1 | 11 |  | 4 | 12 |
|  | 16 |  | 3 | 8 |  | 5 | 9 |

In 13 plots the species was sown as resident of the experimental community (resident populations, K-selection regime), in all other plots it was colonizing and continuously removed by weeding (colonizer populations, r-selection regime). Fewer experimental plots were used for plants grown from seeds because plants of one experimental block were destroyed by mice.
