# Supplementary material for: Density-Independent Mortality and Increasing Plant Diversity Are Associated with Differentiation of Taraxacum officinale into r- and K-Strategists
Source: PLoS One. 2012 Jan 9;7(1):e28121. doi: 10.1371/journal.pone.0028121 (PMC3253783; doi:10.1371/journal.pone.0028121)
Supplement: Table S3 — Measured traits of plants grown from seeds or cuttings. (DOC) [file pone.0028121.s006.doc]

**Table S3**

| Traits | Grown from seeds | | | Grown from cuttings |
| --- | --- | --- | --- | --- |
|  | Offspring 1 | Offspring 2 | Offspring 3 |  |
| Shoot biomass (total) (mg) | × | × | × | × |
| Root biomass (mg) | × | n.a. | n.a. | n.a. |
| Shoot:root ratio | × | n.a. | n.a. | n.a. |
| Vegetative biomass (leaves) (mg) | × | × | × | × |
| Reproductive biomass (stalk, flowers, seeds, buds) (mg) | × | × | × | × |
| Number of leaves | × | × | × | × |
| Length of the longest leaf (cm) | × | × | × | × |
| Number of flower heads | × | × | × | × |
| Flower head diameter (cm) | × | n.a. | n.a. | n.a. |
| Mean seed mass (mg) | n.a. | × | n.a. | × |
| Seed mass per flower head (mg) | n.a. | × | n.a. | × |
| Seed mass per plant (mg) | n.a. | × | n.a. | x |
| Seed number per plant | n.a. | × | n.a. | × |
| First day of flowering* | × | n.a. | n.a. | n.a. |
| Day of seed maturity* | n.a. | × | × | × |
| Height of flower stalk at seed maturity (cm) | n.a. | × | × | × |

Note: n.a. = not measured

* day from 01.03.2008 to 05.04.2008.
