# Supplementary material for: Density-Independent Mortality and Increasing Plant Diversity Are Associated with Differentiation of Taraxacum officinale into r- and K-Strategists
Source: PLoS One. 2012 Jan 9;7(1):e28121. doi: 10.1371/journal.pone.0028121 (PMC3253783; doi:10.1371/journal.pone.0028121)
Supplement: Appendix S1 — The R code for statistical analysis with mixed-effects models. (DOC) [file pone.0028121.s007.doc]

**Appendix S1**

We used R 2.11.1 (R Development Core Team, [http://www.r-project.org](http://www.r-project.org/)) and the R package *lme4* [63] for data analyses.

Abbreviations of variable names are:

Glasshouseblock Experimental blocks in the glasshouse for cultivation of offspring from the biodiversity experiment

Fieldblock Experimental blocks of the biodiversity experiment

Plot Experimental plots of the biodiversity experiment

Family Identity of maternal plants of seedlings

rK Selection regime (r vs. K-selection)

sowndiv Species richness of the experimental plots of the biodiversity experiment

**R code:**

sg0<-lmer(X~1+(1|Glasshouseblock)+(1|Fieldblock)+(1|Fieldblock:Plot)+(1|Fieldblock:Plot:Family),REML=F)

sg1<-lmer(X~1+rK+(1|Glasshouseblock)+(1|Fieldblock)+(1|Fieldblock:Plot)+(1|Fieldblock:Plot:Family),REML=F)

sg2<-lmer(X~1+rK+log(sowndiv)+(1|Glasshouseblock)+(1|Fieldblock)+(1|Fieldblock:Plot)+(1|Fieldblock:Plot:Family),REML=F)

sg3<-lmer(X~1+rK+log(sowndiv)+rK:log(sowndiv)+(1|Glasshouseblock)+(1|Fieldblock)+(1|Fieldblock:Plot)+(1|Fieldblock:Plot:Family),REML=F)

anova(sg0,sg1,sg2,sg3)
